# Ursodeoxycholic Acid Treatment Restores Gut Microbiota and Alleviates Liver Inflammation in Non-Alcoholic Steatohepatitic Mouse Model

**DOI:** 10.3389/fphar.2021.788558

**Published:** 2021-12-06

**Authors:** Hu Li, Qingling Wang, Peizhan Chen, Chenghua Zhou, Xinxin Zhang, Li Chen

**Affiliations:** ^1^ Department of Gastroenterology, Ruijin Hospital, School of Medicine, Shanghai Jiao Tong University, Shanghai, China; ^2^ Department of Infectious Disease, Shanghai Jiao Tong University Affiliated Sixth People`s Hospital, Shanghai, China; ^3^ Department of Infectious Disease, Research Laboratory of Clinical Virology, Ruijin Hospital, Shanghai Jiao Tong University, School of Medicine, Shanghai, China; ^4^ Department of Infectious Disease, The First People’s Hospital of Yunnan Province, Kunming, China; ^5^ Central Laboratory, Ruijin Hospital, School of Medicine, Shanghai Jiao Tong University, Shanghai, China

**Keywords:** non-alcoholic fatty liver disease, gastrointestinal microbiome, gut–liver axis, dysbiosis, therapy

## Abstract

Gut microbiota dysbiosis plays an important role in the progression of non-alcoholic fatty liver disease (NAFLD), and no approved drugs are available for NAFLD treatment. In this study, we aimed to explore the dynamic changes of gut microbiota at the different stages of NAFLD and determine whether ursodeoxycholic acid (UDCA) could improve liver histopathological features of non-alcoholic steatohepatitis (NASH) mice induced by a high-fat high-cholesterol (HFHC) diet and its impact on gut microbiota. 6-week-old male C57BL/6 mice were fed with a HFHC or normal diet for 12, 18, and 24 weeks, respectively, to simulate the different stages of NAFLD. 16s ribosomal RNA genes from mice fecal samples at the different time points were sequenced to evaluate the dynamic changes of the gut microbiota. Then, C57BL/6 mice were fed with a HFHC diet for 24 weeks to establish the NASH model. Different doses of UDCA were administered intragastrically for additional 4 weeks. Normal diet–fed mice were taken as control. Serum samples, liver, and intestine tissues were harvested for biochemical tests and histopathological examinations. 16s ribosomal RNA genes from mice fecal samples were sequenced to assess the structural changes of gut microbiota. HFHC diet–fed mice developed simple steatosis, steatohepatitis, and fibrosis at 12, 18, and 24 weeks, respectively. The profile of gut microbiota dynamically changed with the different stages of NAFLD. NASH mice had significantly higher abundance of Fecalibaculum, *Coriobacteriaceae*_UCG-002, and *Enterorhabdus*, and lower abundance of norank_f_*Muribaculaceae*, Bacteroides, and Alistipes, which were partially restored by UDCA treatment. UDCA treatment significantly attenuated hepatic inflammation of NASH mice as indicated by the sum of ballooning and lobular inflammation of the NALFD activity score (3.2 ± 0.8 vs 1.8 ± 0.8, *p* = 0.029), and partially restored gut microbiota dysbiosis, and increased the expression of Claudin-1 and ZO-1 in the intestine, but did not activate the suppressed Farnesoid X receptor signal pathway. Conclusions: The gut microbiota dynamically changes with the different stages of NAFLD. UDCA treatment (120 mg/kg) could partially restore gut microbiota, repair gut barrier integrity, and attenuate hepatic inflammation in the NASH mouse model.

## Introduction

Non-alcoholic fatty liver disease (NAFLD) is defined as the presence of hepatic steatosis without secondary causes of hepatic fat accumulation, ranging from non-alcoholic fatty liver (NAFL) to non-alcoholic steatohepatitis (NASH) with or without liver fibrosis (Chalasani et al., 2018). NASH is the active subtype of NAFLD and can potentially progress to liver cirrhosis or hepatocellular carcinoma over time (Sheka et al., 2020). Epidemiology studies and meta-analysis showed that the global prevalence of NAFLD is approximately 25%, with the highest rates in the South America and the Middle East, and the lowest in Africa (Younossi et al., 2016; Fan et al., 2017; Sheka et al., 2020). Currently, NASH has been the second leading cause of end-stage liver disease and liver transplantation in the United States and European countries (Noureddin et al., 2018), which imposes a heavy burden on health-care resources and individuals. The prevalence of NAFLD showed rapid growth in China, from 23.8% in the early 2000s to 32.9% in 2018, and the total NAFLD population is estimated to increase to about 300 million cases by 2030 (Zhou et al., 2020).

The pathophysiology of NAFLD is complex, involving lipotoxicity, inflammasome activation, *etc*., and the exact molecular mechanism remains incompletely understood. The “Multiple parallel hits” hypothesis holds that lipotoxicity and gene polymorphism, together with alteration of gut microbiota, contribute to the progression of NAFLD (Tilg et al., 2021). Over the last decade, a growing number of studies support that gut microbiota plays a crucial role in the pathogenesis of NAFLD (Leung et al., 2016; Mehal and Loomba, 2019). Various degrees of gut microbiota dysbiosis exists in NAFLD patients, and patients with different stages of NAFLD often present with variable microbial signatures. Data regarding the gut microbiota dysbiosis in NAFLD are discordant across studies due to the variability of patients, NAFLD diagnostic methods, and sequencing tools (Aron-Wisnewsky et al., 2020). However, whether the gut microbiota is a primary pathogenic factor still needs further study. Thus, demonstrating the causality of gut microbiota in the development and progression of NAFLD will help identify novel therapeutic targets for treating it.

Currently, no approved drugs are available for NAFLD treatment despite great efforts on new drug discovery in recent years. Weight loss due to lifestyle modifications or physical activity is the cornerstone treatment for NAFLD and the only proven method to improve the histopathological features of NASH (Chalasani et al., 2018). A meta-analysis including eight randomized controlled studies (RCTs) demonstrated that at least 5% body weight loss was associated with improvement in hepatic steatosis, whereas ≥7% reduction of body weight could improve histological disease activity (Musso et al., 2012). However, less than 30% NAFLD patients could achieve this goal (Vilar-Gomez et al., 2015), suggesting that pharmacological therapies remain unmet medical needs.

Ursodeoxycholic acid (UDCA) is a hydrophilic, non-toxic, secondary bile acid in humans and predominantly used to treat cholestatic liver diseases, such as primary biliary cholangitis. As its immunomodulatory, anti-apoptotic, and anti-oxidant activities in the liver, UDCA has also been empirically applied to the treatment of NAFLD, but data on the efficacy are controversial. Animal studies have shown that UDCA treatment could attenuate hepatic steatosis, lobular inflammation, and even reverse liver fibrosis in NAFLD (Namisaki et al., 2016; Carino et al., 2019), but RCTs in human study failed to improve the overall histology in NASH patients (Leuschner et al., 2010; Ratziu et al., 2011). The inconsistent results across the studies are likely attributable to the different dosages of UDCA used or the species differences between humans and rodents in the response to UDCA treatment. Therefore, the present study aimed to assess the effects of dose-response of UDCA on the NASH induced by a high-fat high-cholesterol diet (HFHC) and its influence on the gut microbiota in NASH mice.

## Materials and Methods

### Animals and Treatment

Male C57BL/6 mice (4 weeks of age) were purchased from Shanghai SLAC Laboratory Animal Co., Ltd. (Shanghai, China) and housed in specific pathogen-free conditions on a 12-h light/dark cycle with free access to water and food. After acclimating to the housing environment for 2 weeks, mice (6 weeks of age) were fed a HFHC diet (D09100310, Research Diet, United States, containing 40 kcal% fat, 20 kcal% fructose, and 2% cholesterol) for 12, 18, and 24 weeks, respectively, to simulate the different stages of NAFLD. NASH mouse models were established by feeding a HFHC diet for 24 weeks and then treated with UDCA (MB5752, MeilunBio, China) while continuing to receive the HFHC diet for additional 4 weeks. The mice in the control groups were fed a standard normal diet (SLACOM, China, containing 20.5% crude protein, 4% crude fat, 5% crude fiber, 8% crude ash, and 10% water). UDCA was dissolved in 0.5% carboxymethyl cellulose sodium (CMC) with a concentration of 3 mg/ml, 6 mg/ml, and 12 mg/ml, respectively, and then administered daily by gavage (30 mg/kg, 60 mg/kg, or 120 mg/kg) for 4 weeks after 24 weeks of the HFHC diet. The volume of gavage was calculated according to the body weights of mice. Mice body weights were measured once a week during the experiments. Mice were anesthetized with 2% phenobarbital sodium (50 mg/kg) by intraperitoneal injection after fasting overnight for 24 h after the last UDCA treatment. The whole blood was withdrawn by cardiac puncture, allowed to clot for at least 30 min, and centrifuged at 3,000 rpm for 10 min to obtain serum. After being sacrificed by dislocation of the cervical vertebra, mice were fixed in the dissecting tray. The abdominal cavity of mice was opened, and the liver was exposed, the inferior vena cava was cut off, the blood was sucked up using absorbent paper, and then the whole liver was separated and removed using tweezers, measured, and the liver weight was recorded. A part of the liver and intestine was fixed in 4% neutral buffered formalin for histopathological analysis, and the others were frozen swiftly in liquid nitrogen. About 3 cm of the distal ileum was harvested for histopathological and immunohistochemistry analyses. All animal experiments were performed with the approval of the Institutional Animal Care and Use Committee of Ruijin Hospital, Shanghai Jiao Tong University School of Medicine.

### Liver and Intestine Histopathological Analysis

Mouse hepatic and small intestinal samples were fixed in 4% neutral buffered formalin and embedded in paraffin. Paraffin-embedded samples were sectioned and then subjected to hematoxylin and eosin (H&E) staining for assessment of liver and intestine histopathology. Oil Red O staining was performed on the frozen sections to evaluate the hepatic steatosis. Paraffin sections were stained with Masson’s trichrome to evaluate the degree of liver fibrosis. The NAFLD activity score (NAS) was calculated from the grade of steatosis, inflammation, and ballooning. In brief, steatosis was quantified as 0 (<5%), 1 (5–33%), 2 (>33–66%), and 3 (>66%) based on the percentage of hepatocytes containing fat droplets. Ballooning degeneration was scored as 0 (none), 1 (few), or 2 (many) according to the amount of ballooning hepatocytes. Lobular inflammation was scored as 0 (no foci), 1 (<2 foci), 2 (2–4 foci), and 3 (>4 foci) according to the inflammation foci per 200× field (Kleiner et al., 2005). The Chiu scoring system was used to evaluate the intestinal histopathologic findings (Chiu et al., 1970). The hepatic and intestinal histology and NAS score were evaluated by a pathologist, who was blinded to the groups.

### Serum Lipopolysaccharides and Cytokines Analysis

The levels of serum lipopolysaccharides (LPS) and interleukin (IL)-4 were measured using mouse LPS enzyme-linked immunosorbent assay (ELISA) technique kits (CSB-E E13066m, CUSABIO, United States) and mouse IL-4 ELISA kit (EK204/2, DAKEWE, China), respectively. The levels of serum tumor necrosis factor (TNF)-α, IL-1β, and IL-10 were measured using a Multi-Analyte Flow Assay Kit (740621, BioLegend, San Diego, America) according to the manufacturer protocols.

### Liver Tissue RNA Extraction and Quantitative Real-Time Polymerase Chain Reaction

The total RNA was extracted from mice livers using a TRIzol Reagent Kit (R0016, Beyotime Biotechnology, China). 1 μg of total RNA was reverse-transcribed to cDNA using PrimeScript™ RT Master Mix (RR036Q, Takara Bio, Japan) according to the manufacturer’s instruction. Quantitative real-time polymerase chain reaction (RT-qPCR) was performed using a QuantiNova SYBR Green PCR Kit (208054, QIAGEN, Germany) and Light Cycler^®^ 96 Real-time PCR system (Roche, Switzerland). The Gapdh gene was used as an internal control. The primers used in this study are listed in Supplementary File S1. The relative expression of mRNA was calculated using the 2^−ΔΔCt^ method.

### Small Intestine Immunohistochemistry Staining

Intestinal barrier function was evaluated using immunohistochemistry staining. Briefly, paraffin sections of mice small intestine were deparaffinized with xylene and gradient ethanol solutions, and then treated with a series of antigen recovery using 0.01 M sodium citrate-hydrochloric acid buffer solutions. The small intestine sections were incubated with anti–ZO-1 polyclonal antibody (1:500, PA5-85256, ThermoFisher scientific, United States) and anti–Claudin-1 polyclonal antibody (1:400, 71–7,800, ThermoFisher scientific, United States) primary antibody at 4°C overnight after treatment with 3% hydrogen peroxide for 25 min at room temperature. Then, the small intestine sections were incubated with horseradish peroxidase (HRP)-conjugated secondary antibody (1:200, GB23303, Servicebio, China) for 50 min at room temperature, following 3,3′-diaminobenzidine–based HRP reaction. The small intestine sections were counterstained with hematoxylin and examined using a COIC XSP-204 microscope (COIC, China). Immunohistochemistry results were evaluated using the H score ranging from 0 to 300 (Specht et al., 2015).

### Fecal DNA Extraction and 16s Ribosomal RNA Gene Sequencing

About 5–6 fecal pellets were collected at about 8:00 am and stored at −80°C until further processing with E.Z.N.A.^®^ Soil DNA Kit (D5625, OMEGA bio-tek, United States). The concentration of extracted DNA was measured using Nanodrop 2000 (Thermo Fisher Scientific, United States). The V3–V4 region in the bacterial 16S rRNA gene was amplified by PCR with the primer pair (338F: 5′ACT​CCT​ACG​GGA​GGC​AGC​AG3`; 806R 5′GGACTACHVGGGTWTCTAAT3′), using TransStart^®^ FastPfu DNA polymerase (AP221-01, TransGen, China). PCR products were examined using 2% agarose gel electrophoresis and then purified using an AxyPrep DNA Gel Extraction Kit (AP-GX-500, Axygen, United States). The PCR products were adequate for sequencing (Supplementary File S2). Purified PCR products were quantified using a Quantus™ Fluorometer (Promega, United States) and pooled in the same concentration. Paired-end sequencing was performed on the Miseq PE300 platform (Illumina, United States).

### Microbial Analysis

Fastq software (Chen et al., 2018) was used for quality control of raw Illumina sequencing data. Sequencing reads were assembled using FLASH software (Magoc and Salzberg, 2011). Operational taxonomic units (OTUs) were clustered at the 97% similarity level using UPARSE software (Edgar, 2013) after removing the chimeric sequences. The identified taxonomy was aligned and annotated using the Silva 16S database (V138) and RDP classifier (Wang et al., 2007) setting the contrast thresholds of 70%. Shannon and rarefaction curves were performed on the OTU table to detect the sequencing depth. α-diversity, including Sobs index, Shannon index, ACE index, and Chao1 index, was calculated from the OTU table to compare the richness and evenness of the gut microbiota among the different groups. β-diversity was estimated using Bray-Curtis distance based on principal coordinate analysis, non-metric multidimensional scale analysis, and principal component analysis as appropriate.

### Statistical Analysis

All data were expressed as mean ± standard deviation (SD) and analyzed using appropriate statistical methods with GraphPad Prism software (version 8.2.1). Two-tailed Student’s *t-*test was used to analyze the differences between two groups, while one-way analysis of variance (ANOVA) was used to analyze the differences among three or more groups, and Bonferroni’s *post hoc* test was applied for multiple comparisons. A *p*-value of less than 0.05 was considered statistically significant.

## Results

### High-Fat High-Cholesterol Diet Induces Non-Alcoholic Fatty Liver Disease Development

**FIGURE 1:**
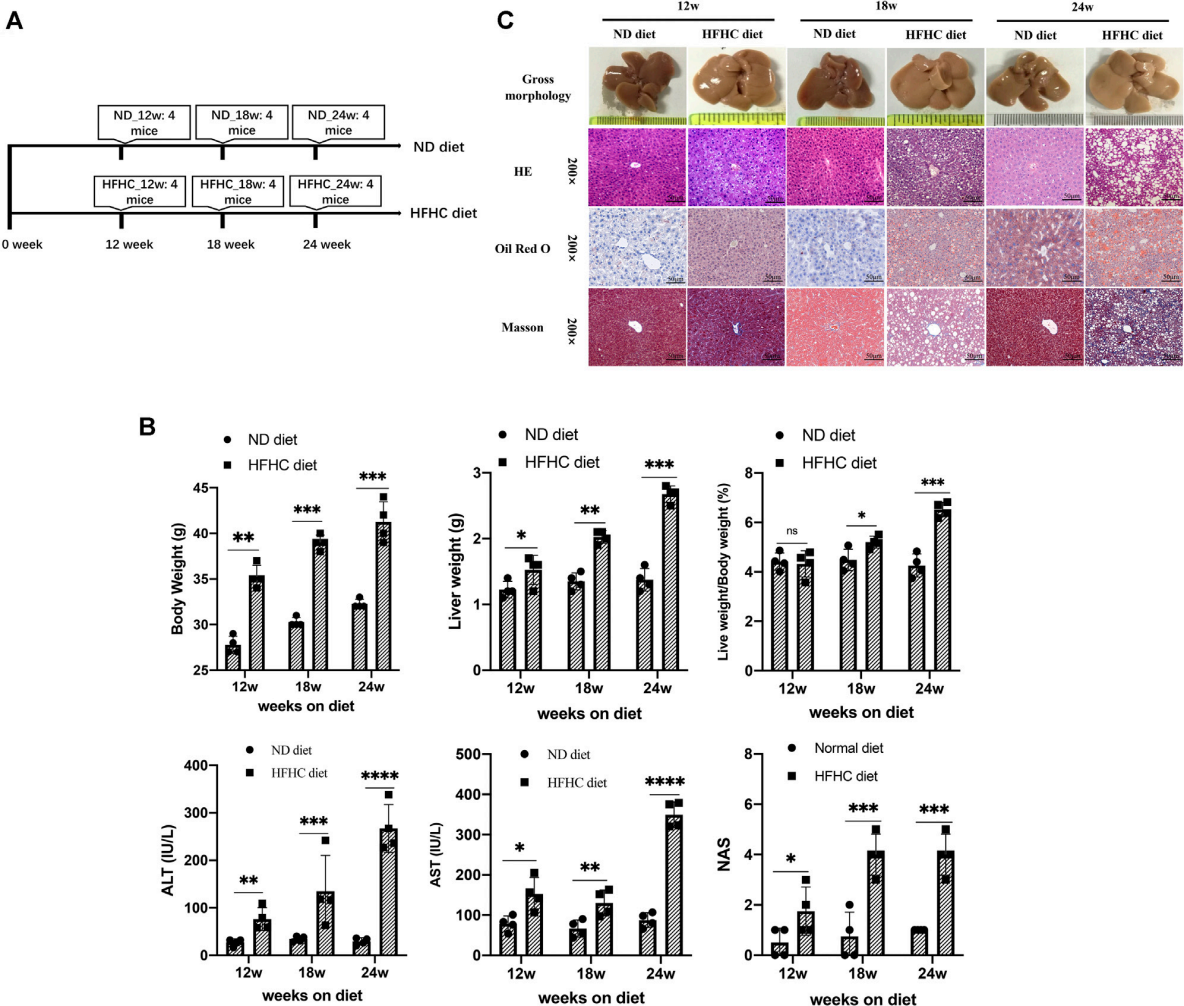
Establishment of a mouse model of HFHC-diet–induced NAFLD. **(A)** Schematic overview of experimental design (*n* = 4 for each group). **(B)** Body weights, liver weights, liver/body weight ratio, serum ALT and AST, and NAS scores in mice fed on ND or HFHC diet for 12, 18, and 24 weeks. **(C)** Gross morphology, H&E, Oil red O and Masson`s trichrome staining of hepatic tissues from ND or HFHC-fed mice. Values represent mean ± standard deviation with *p < 0.05, **p < 0.01, ***p < 0.001, ****p < 0.000 and ns vs HFHC_12w group. Abbreviation: HFHC, high-fat high-cholesterol; NAFLD, non-alcoholic fatty liver disease; ALT, alanine transaminase; AST, aspartate transaminase; NAS, NAFLD activity score; ND, normal diet; ns, not significant.

To simulate the different stages of human NAFLD, mice were fed a HFHC diet for 12, 18, and 24 weeks, respectively, whereas the mice in the control group were fed a normal chow diet and taken as control (Figure 1A). Mice in the HFHC group had faster body weight gains than those in the control group. The liver weights (LWs) and body weights (BWs) of mice in the HFHC group significantly increased over time, and there were significant differences regarding the mice liver weights and liver weight to body weight ratios at the 18th and 24th weeks between the HFHC and control groups. The serum levels of alanine transaminase (ALT) and aspartate transaminase (AST) were significantly elevated in HFHC diet–fed mice compared to ND diet–fed mice, and the differences reached a statistical significance from the 12th week onward (*p* < 0.05). Consistently, the NAS scores in HFHC diet–fed mice increased over time and were significantly higher than those in ND diet–fed mice at the 12th, 18th, and 24th weeks (*p* < 0.01) (Figure 1B).

As shown in Figure 1C, H&E and Oil Red O staining demonstrated mild hepatocyte steatosis without ballooning degeneration and lobular inflammation in HFHC diet–fed mice at 12 weeks. At 18 weeks, mice liver histology was characterized by significant hepatocytes macrovesicular steatosis, slight ballooning degeneration, and lobular inflammation, but without perisinusoidal fibrosis. At 24 weeks, macrovesicular steatosis, ballooning degeneration, and lobular inflammation were common as revealed by H&E and Oil Red O staining, and mild-to-moderate perisinusoidal fibrosis was observed using Masson’s trichrome staining.

### Dynamic Changes of Gut Microbiota Profile at the Different Stages of Non-Alcoholic Fatty Liver Disease Development

**FIGURE 2:**
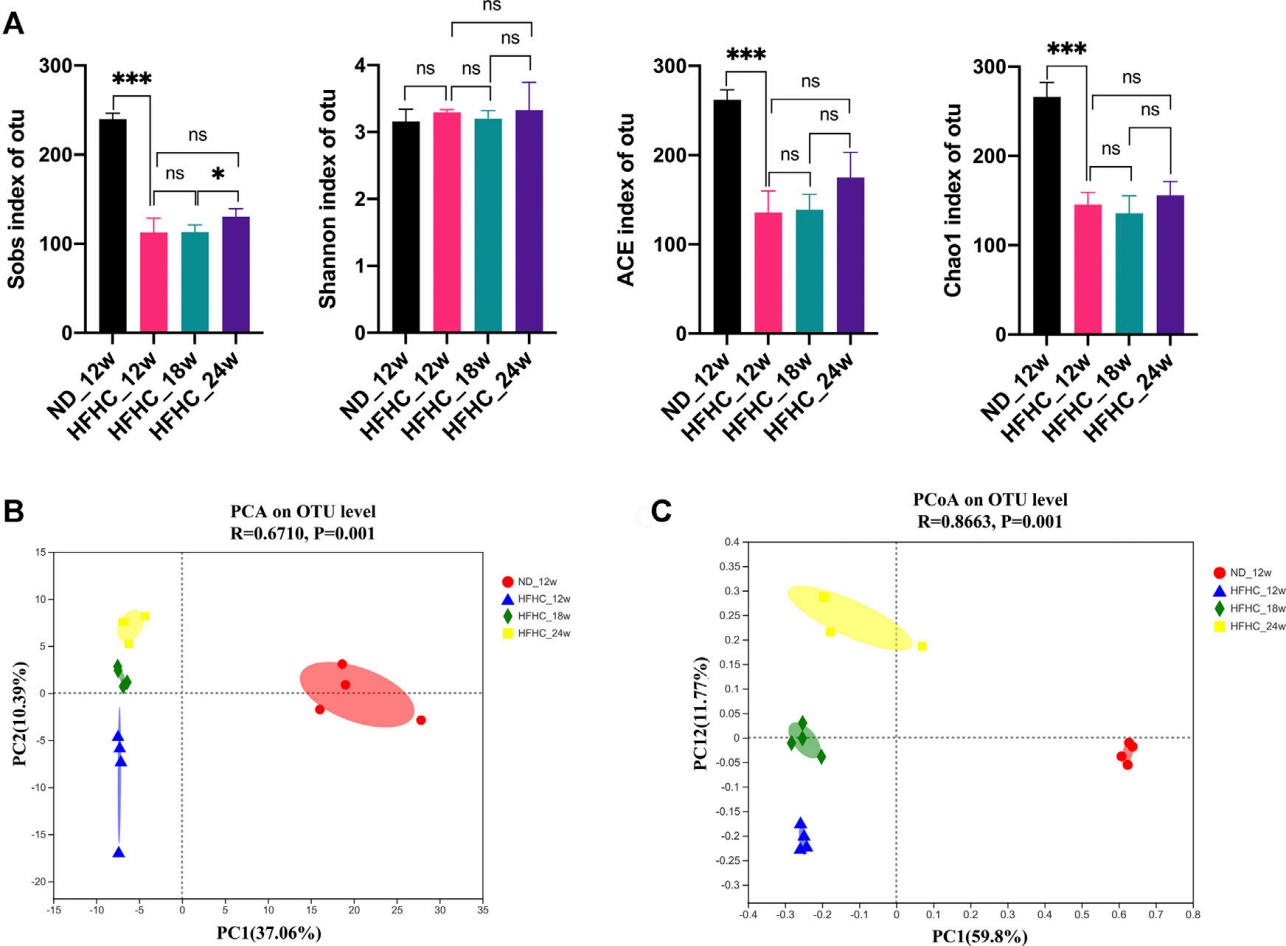
α-diversity and β-diversity analyses of the gut microbiota in mice at the different stages of NAFLD. **(A)** Sobs index, Shannon index, ACE index, and Chao1 index on the OTU level. **(B)** PCA analysis. **(C)** PCoA analysis based on Bray-Curtis distance. Values represent mean ± standard deviation with *p < 0.05, ***p < 0.001 and ns vs HFHC_12w group. Abbreviation: NAFLD, non-alcoholic fatty liver disease; OTU, Operational taxonomic unit; PCA, principal component analysis; PCoA, principal coordinate analysis; HFHC, high-fat high-cholesterol; ns, not significant.

To assess the dynamic changes of the gut microbiota at the different stages of NAFLD, the V3–V4 regions of gut bacterial 16S rRNA genes from HFHC diet–fed mice at weeks 12, 18, and 24 and from ND fed–mice at week 12 were sequenced using the next-generation sequencing. A total of 829 296 high-quality reads were obtained from 16 samples, with an average of 81 831 ± 5,017 reads per sample. A total of 413 OTUs were clustered from these reads based on a 97% sequence similarity level. Shannon and Rarefaction curves showed that the current sequencing depths were sufficient to capture the majority of gut microbial in all samples (Supplementary File S3). As shown in Figure 2A, the Sobs index, ACE index, and the Chao1 index in the HFHC_12w group were significantly lower than the ND_12w group (*p* < 0.001), but there were no significant differences in the Sobs index, Shannon index, ACE index, and the Chao1 index among the HFHC_12w, the HFHC_18w, and HFHC_24w groups. Principal component analysis (PCA) and Bray-Curtis distance–based principal coordinate analysis (PCoA) were used to analyze the structure change of the gut microbiota from each sample based on the relative abundance of OTUs, and the results showed that the gut microbiota of HFHC-fed mice was completely separated from that of ND-fed mice. In addition, analysis of similarities of PCA and PCoA indicated a significant separation among the gut microbiota of HFHC_12w, HFHC_18w, and HFHC_24w groups (*p* < 0.05) (Figures 2B,C).

**FIGURE 3:**
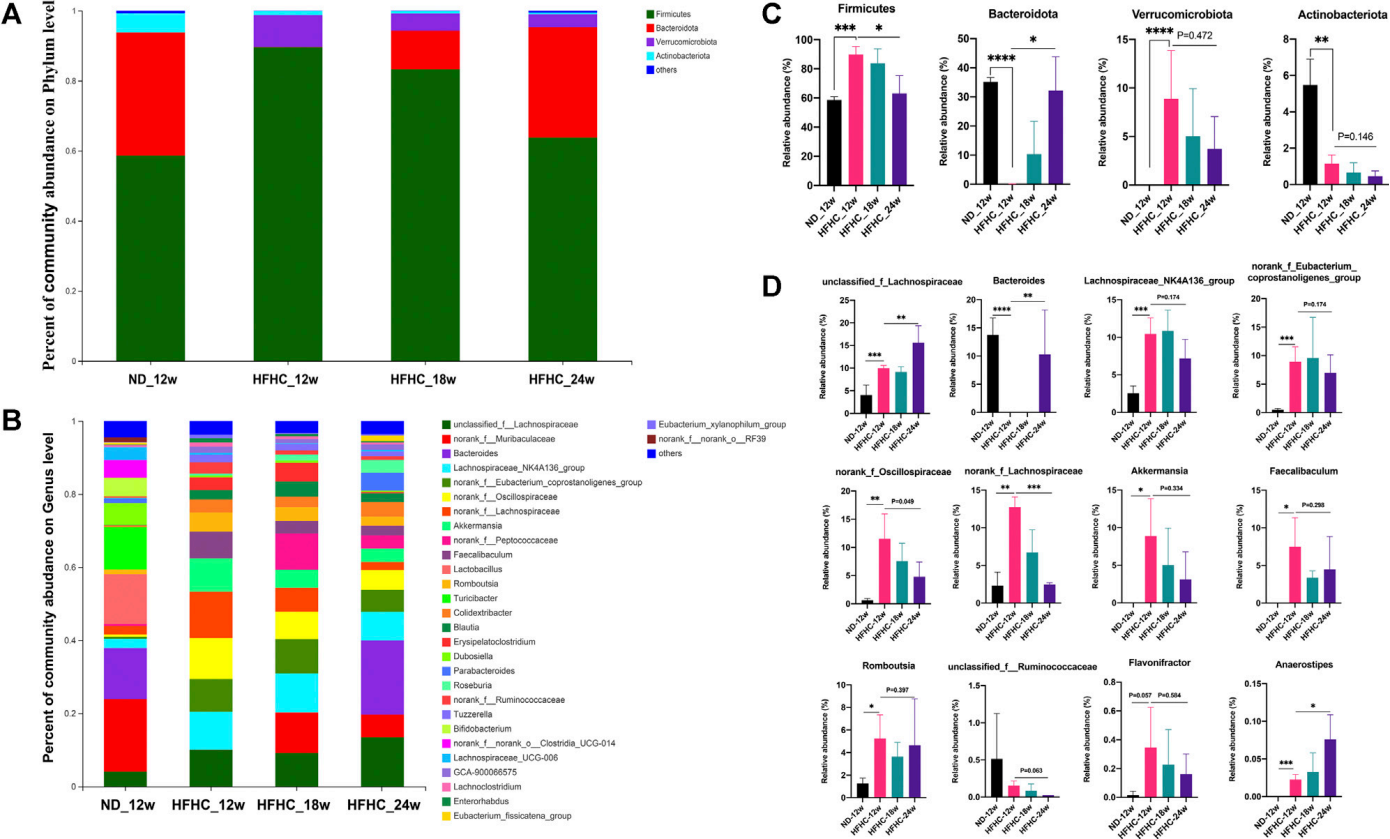
Altered gut microbiome at the different stages of NAFLD. Relative abundance of bacterial communities from mice at the different stages of NAFLD at the phylum **(A)** and genus level **(B)**. Comparison of average abundance of specific gut bacteria from mice at the different stages of NAFLD at the phylum **(C)** and genus level **(D)**. Values represent mean ± standard deviation with *p < 0.05, **p < 0.01, ***p < 0.001 and ****p < 0.000 vs HFHC_12w group. Abbreviation: NAFLD, non-alcoholic fatty liver disease; HFHC, high-fat high-cholesterol.

The relative abundance of the gut bacteria at the phylum and genus levels in each group is presented in Figures 3A,B. At the phylum level, mice in the HFHC_12w group had an increased relative abundance of Firmicutes (85.5 vs 55.6%, *p* = 0.007) and Verrucomicrobiota (7.87 vs 0.002%, *p* = 0.001) and decreased relative abundance of Bacteroidetes (5.0 vs 38.5%, *p* = 0.001), Actinobacteriota (1.2 vs 5.2%, *p* < 0.001), and Proteobacteria (0.06 vs 0.53%, *p* = 0.003) compared to those in the ND_12w group. However, the relative abundance of Firmicute, Verrucomicrobiota, and Actinobacteriota was decreased stepwise, whereas the relative abundance of Bacteroidetes was increased stepwise among the HFHC_12w, HFHC_18w, and HFHC_24w groups (Figure 3C). At the genus level, the relative abundance of unclassified_f_Lachnospiraceae, Bacteroides, and Anaerostipes was increased, while that of norank_f_Oscillospiraceae, norank_f_Lachnospiraceae and Akkermansia was decreased in a stepwise manner during the progression of NAFLD (Figure 3D).

### Ursodeoxycholic Acid Ameliorated Hepatic Inflammation in the High-Fat High-Cholesterol–Diet–Induced Non-Alcoholic Steatohepatitis Model

**FIGURE 4:**
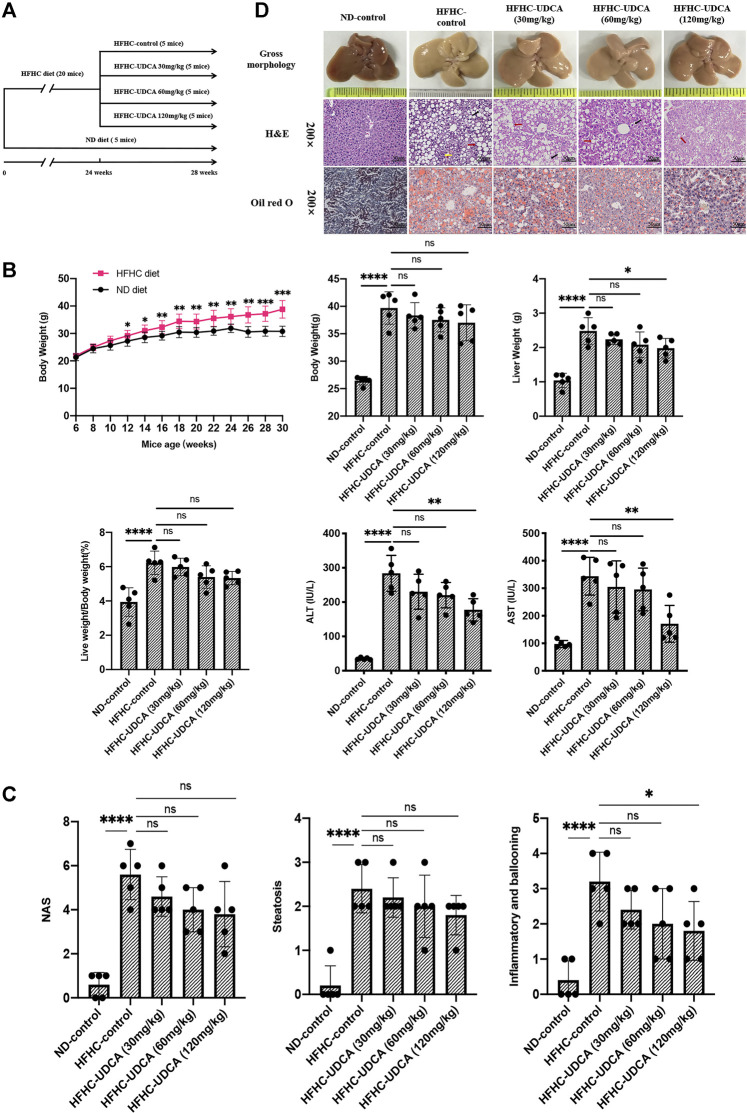
UDCA ameliorates hepatic inflammation in NASH independent of steatosis. **(A)** Research scheme of UDCA in the treatment of HFHC-diet–induced NASH (*n* = 5 for each group). **(B)** UDCA treatment significantly lowered serum ALT and AST levels of NASH mice but not body weight and liver weight. **(C)** UDCA treatment ameliorated hepatic inflammation in NASH mice in a dose-dependent pattern but not steatosis. **(D)** Gross morphology, H&E, and Oil red O staining of hepatic tissue from mice in ND-control group, HFHC-control group, and different doses of UDCA treatment group. Values represent mean ± standard deviation with *p < 0.05, **p < 0.01, ****p < 0.000 and ns vs HFHC-control group. Abbreviation: UDCA, ursodeoxycholic acid; NASH, non-alcoholic steatohepatitis; HFHC, high-fat high-cholesterol; ALT, alanine transaminase; AST, aspartate transaminase; ND, normal diet; ns, not significantly.

To evaluate the effects of UDCA treatment on NASH, HFHC diet–fed mice were administered with different doses of UDCA (30, 60, and 120 mg/kg/d) or CMC (HFHC-control group) at week 24, and an additional HFHC diet for 4 weeks (Figure 4A). Normal diet–fed mice were taken as ND control. After 28 weeks of HFHC feeding, the LW and LW/BW ratios of the HFHC-fed mice were significantly increased compared to those of the ND-fed mice. However, the LW and LW/BW ratios did not differ between the UDCA and vehicle treatment groups after 28 weeks of HFHC feeding. Compared to the HFHC-control mice, HFHC-fed mice treated with UDCA for 4 weeks exhibited lower serum ALT and AST levels in a dose-dependent pattern and reached a significant difference in the 120 mg/kg UDCA treatment group (Figure 4B). HFHC-fed mice had significantly higher levels of serum total cholesterol and blood glucose and lower level of triglycerides, but UDCA treatment did not improve these parameters (Supplementary File S4).

Liver histopathology demonstrated that 120 mg/kg UDCA treatment for 4 weeks significantly alleviated hepatic inflammation as indicated by the sum of ballooning and lobular inflammation of NAS compared to the HFHC-control group (3.2 ± 0.8 vs 1.8 ± 0.8, *p* = 0.029). However, improvement in hepatic steatosis was not observed after UDCA treatment for 4 weeks (Figures 4C,D).

### Ursodeoxycholic Acid Treatment Partially Restored the Dysbiosis of the Gut Microbiota in Non-Alcoholic Steatohepatitis Mice Induced by High-Fat High-Cholesterol Diet

**FIGURE 5:**
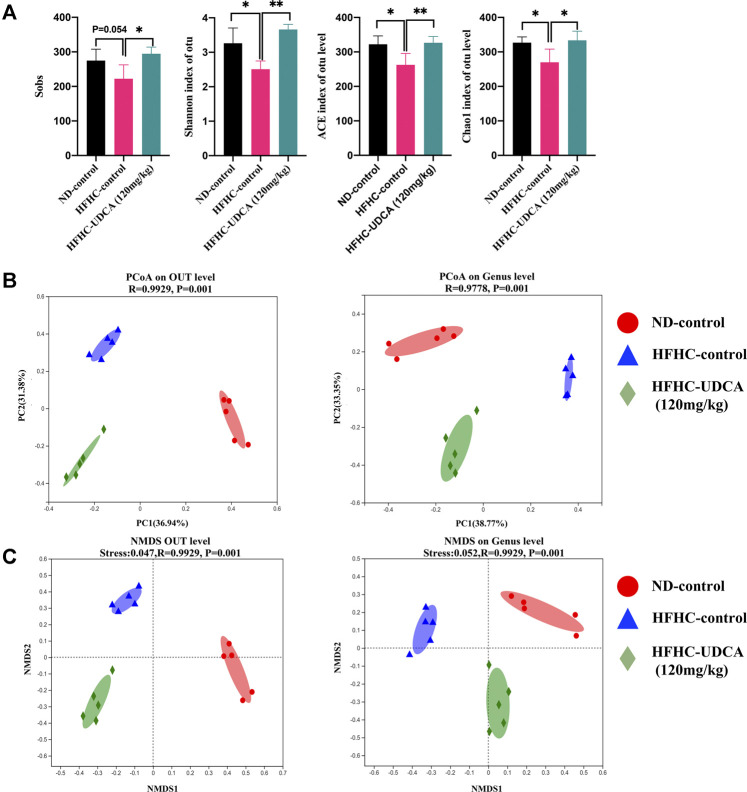
α-diversity and β-diversity analysis of gut microbiota in mice from ND-control, HFHC-control and HFHC-UDCA (120 mg/kg) group. **(A)** Sobs index, Shannon index, ACE index, and Chao1 index on the OTU level. **(B)** PCoA analysis. **(C)** NMDS analysis based on Bray-Curtis distance. Values represent mean ± standard deviation with *p < 0.05 and **p < 0.01 vs HFHC-control group. Abbreviation: ND, normal diet; HFHC, high-fat high-cholesterol; UDCA, ursodeoxycholic acid; OTU, Operational taxonomic unit; PCoA, principal coordinate analysis; NMDS, non-metric multidimensional scale analysis.

To explore the effect of UDCA treatment on the gut microbiota in NASH mice, the V3–V4 regions of gut bacterial 16S rRNA genes from mice in ND-control, HFHC-control, and HFHC-UDCA (120 mg/kg) were sequenced using the next-generation sequencing. A total of 739,585 reads were retrieved. Five hundred ninety-one OTUs were clustered based on the 97% sequence similarity level. Shannon and Rarefaction curves indicated that the sequencing depths were sufficient to reflect the microbiota profile in all samples (Supplementary File S5). The Sobs index, Shannon index, ACE index, and Chao1 index in the HFHC-control group were lower than those in the ND-control group, and UDCA treatment for 4 weeks increased these indexes, suggesting that UDCA treatment could restore the diversity of the gut microbiota from NASH mice (Figure 5A). Bray-Curtis distance based on PCoA analysis showed that the identified gut microbiota were separated into three distinct groups, which was confirmed by non-metric multidimensional scale analysis (NMDS) (Figures 5B,C).

**FIGURE 6:**
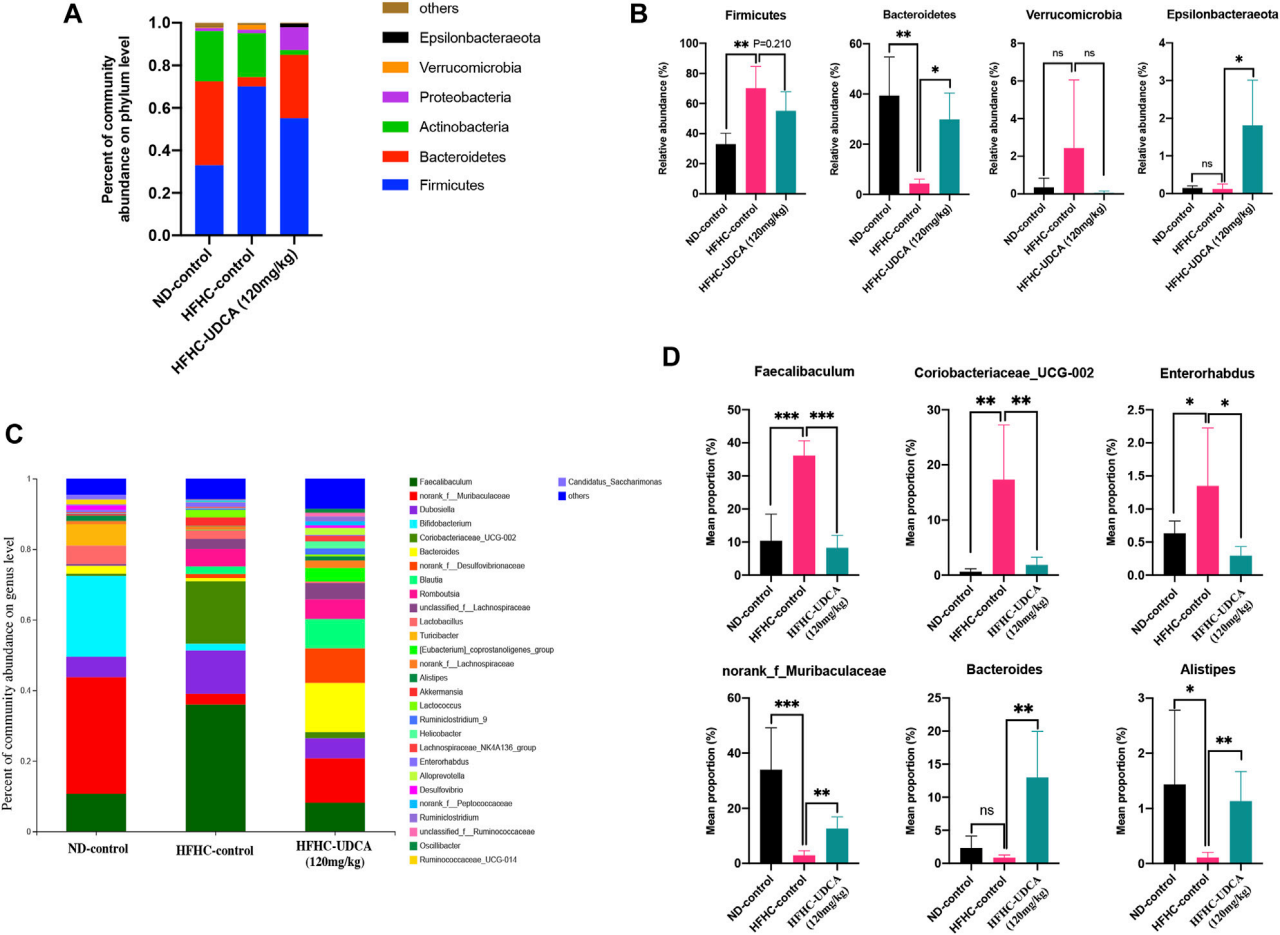
UDCA partially restored the gut microbiome in NASH mice. Relative abundance of bacterial communities from mice in ND-control, HFHC-control and HFHC-UDCA (120 mg/kg) group at the phylum **(A)** and genus level **(C)**. Comparison of average abundance of certain gut bacteria from mice in ND-control, HFHC-control and HFHC-UDCA (120 mg/kg) group at the phylum **(B)** and genus level **(D)**. Values represent mean ± standard deviation with *p < 0.05, **p < 0.01, ***p < 0.001 and ns vs HFHC-control group. Abbreviation: UDCA, ursodeoxycholic acid; NASH, non-alcoholic steatohepatitis; ND, normal diet; HFHC, high-fat high-cholesterol; ns, not significant.

As shown in Figure 6, at the phylum level, the HFHC-control mice had significantly increased relative abundance of Firmicutes and decreased relative abundance of Bacteroidetes compared to the ND-control mice, which were partially restored by UDCA treatment. At the genus level, the HFHC-control mice had a significantly higher abundance of Fecalibaculum, Coriobacteriaceae_UCG-002, and Enterorhabdus, and lower abundance of norank_f_Muribaculaceae, Bacteroides, and Alistipes, which were also partially restored by UDCA treatment.

Phylogenetic Investigation of Communities by Reconstruction of Unobserved States (PICURSt2) (Douglas et al., 2020) was used for microbial community metagenome prediction. At last, a total of 117 differential pathways were identified, such as primary bile acid biosynthesis (Supplementary File S6).

### Ursodeoxycholic Acid Protected Against Intestinal Barrier Disruption and Reduces Serum Lipopolysaccharides and Inflammatory Cytokines in Non-Alcoholic Steatohepatitis Mice

**FIGURE 7:**
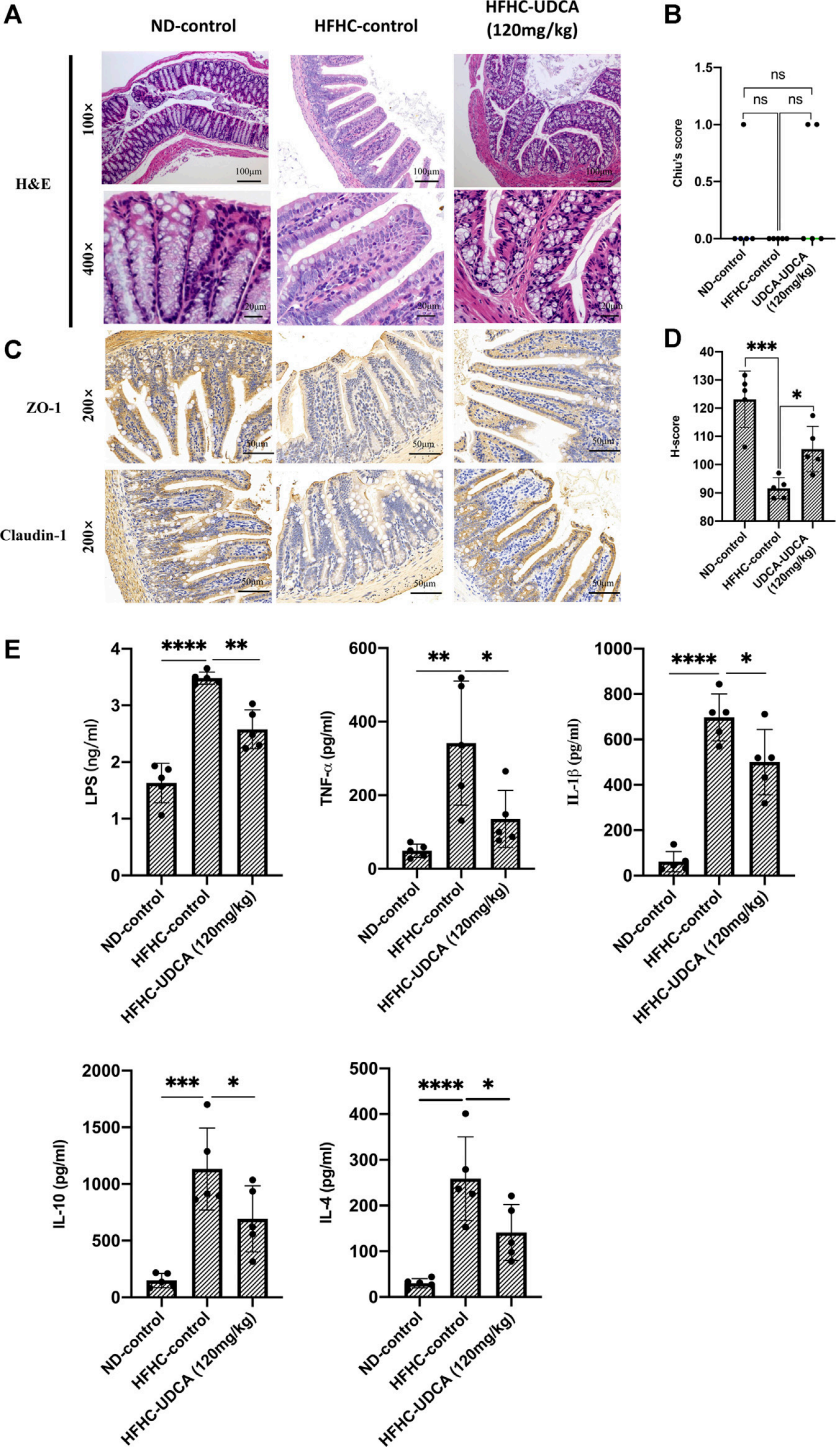
UDCA protects against intestinal barrier disruption and reduces serum LPS and inflammatory cytokines in NASH mice. **(A)** H&E staining of mouse intestinal tissue from ND-control, HFHC-control and HFHD-UDCA (120 mg/kg) group. **(B)** Comparison of the Chiu score for mouse intestinal tissue from ND-control, HFHC-control, and HFHD-UDCA (120 mg/kg) groups. **(C)** Immunohistochemistry staining for ZO-1 and Claudin-1 expression in the mouse intestine from ND-control, HFHC-control, and HFHD-UDCA (120 mg/kg) groups. **(D)** Comparison of H-score for the immunohistochemical evaluation of ZO-1 and Claudin-1 expression in the mouse intestine from ND-control, HFHC-control, and HFHD-UDCA (120 mg/kg) groups. **(E)** UDCA treatment reduces serum LPS and inflammatory cytokines in NASH mice. Values represent mean ± standard deviation with *p < 0.05, **p < 0.01, ***p < 0.001 and ns vs HFHC-control group. Abbreviation: UDCA, ursodeoxycholic acid; LPS, lipopolysaccharide; NASH, non-alcoholic steatohepatitis; HFHC, high-fat high-cholesterol; ND, normal diet; ns, not significant.

As demonstrated above, UDCA treatment could reduce the serum ALT and AST levels, and improve hepatic inflammation in a dose-dependent pattern; thus, we further evaluated the effect of UDCA on the intestinal barrier function and levels of serum endotoxemia and inflammatory cytokines in NASH mice. As shown in Figures 7A–D, no signs of intestinal mucosal inflammation or impairment of the intestinal epithelium integrity were observed in HFHC-fed mice as revealed by H&E staining compared to the ND-fed mice, and UDCA treatment for 4 weeks did not seem to alter the intestinal mucosal structure either. We then evaluated the expression of tight junction protein in the intestinal epithelium. Immunohistochemistry results suggested that the expressions of Claudin 1 and ZO-1 in the HFHC-control mice were significantly decreased compared to those of ND-control mice, but increased after 120 mg/kg of UDCA treatment.

Thereafter, we evaluated the effect of UDCA treatment on serum LPS and inflammatory cytokines (TNF-α, IL-1β, IL-10, and IL-4). As shown in Figure 7E, serum LPS, TNF-α, IL-1β, IL-10, and IL-4 levels in HFHC-control mice were significantly elevated compared to the ND-control mice, but significantly decreased after 120 mg/kg of UDCA treatment.

### Effect of Ursodeoxycholic Acid Treatment on Bile Acid–Related Gene Expression

**FIGURE 8:**
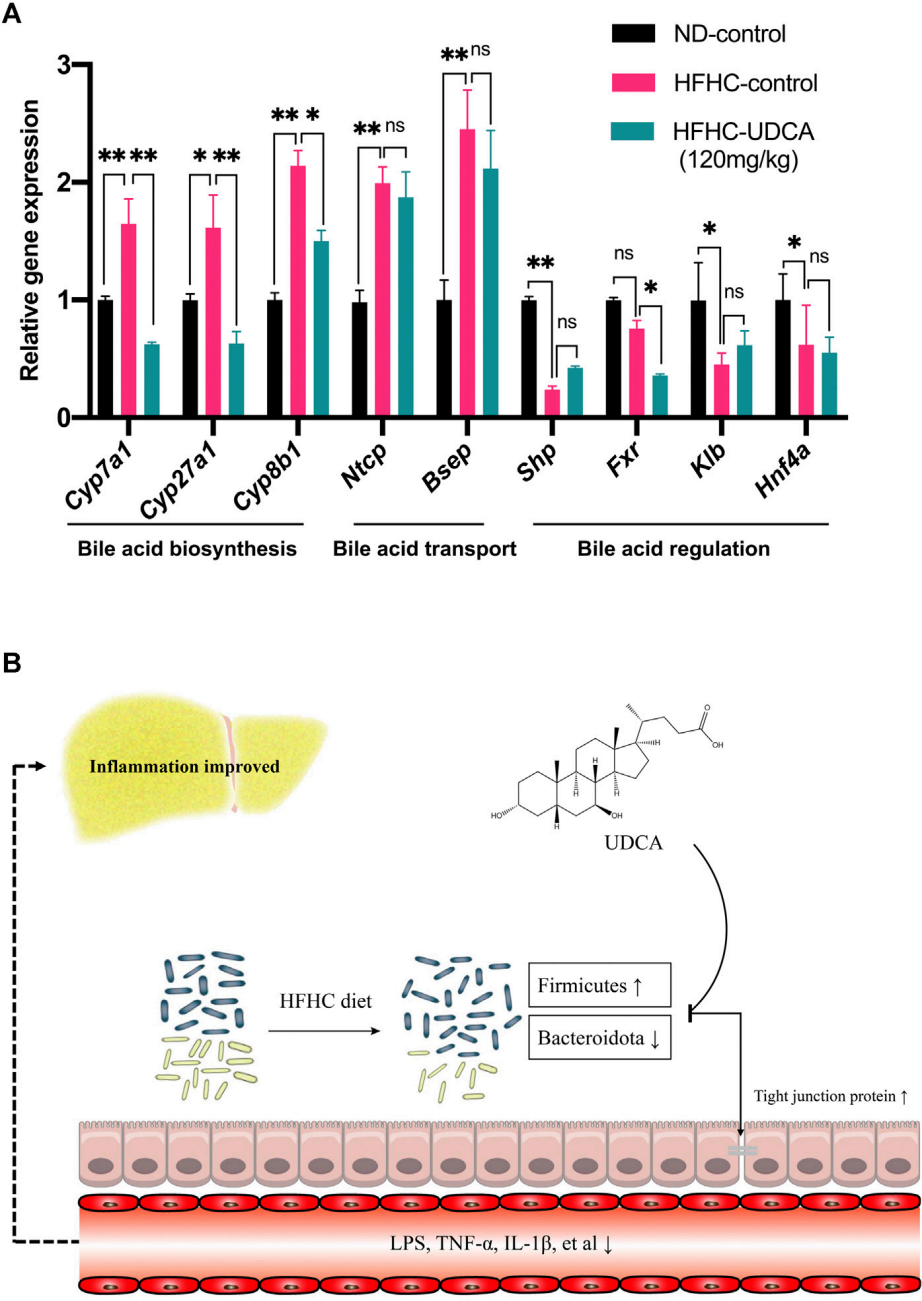
Effect of UDCA treatment on hepatic bile acid–related genes expression in NASH mice. **(A)** UDCA treatment did not activate the suppressed FXR signal pathway in NASH mice. **(B)** Proposed mechanism of UDCA treatment alleviating hepatic inflammation in NASH mice. Values represent mean ± standard deviation with *p < 0.05, **p < 0.01, and not significant (ns) vs HFHC_12w group. Abbreviation: UDCA, ursodeoxycholic acid; NASH, non-alcoholic steatohepatitis; FXR, farnesoid X receptor; HFHC, high-fat high-cholesterol.

Expressions of genes related to bile acid metabolism in mice livers were investigated using RT-qPCR among the ND-control, HFHC-control, and HFHC-UDCA (120 mg/kg) groups. The expression of genes related to bile acid biosynthesis, such as Cyp7a1, Cyp27a1, and Cyp8b1, was significantly increased in NASH mice lives, and so did the bile acid transportation–related genes (Ntcp and Bsep). UDCA treatment reversed the expression of bile acid biosynthesis–related genes, but not those bile acid transportation–related genes. In contrast, NASH mice had decreased expression of Shp, Fxr, Klb, and Hnf4α, which were genes related to the regulation of the bile acid metabolism. However, UDCA treatment did not reverse the expression of these genes (Figure 8A).

## Discussion

In the present study, we have successfully established a NAFLD mice model fed with a HFHC diet which demonstrated pathologically NAFL, NASH, and NASH with fibrosis at 12, 18, and 24 weeks, respectively. 16S rRNA gene sequencing showed the dynamic changes of the gut microbiota during the NAFLD progression from NAFL to NASH and NASH with fibrosis. Moreover, we have also shown that UDCA could ameliorate hepatic inflammation, but not steatosis, in a dose-dependent pattern and partially restore the dysbiosis of the gut microbiota for the treatment of NASH. In addition, UDCA treatment could protect against intestinal barrier disruption and reduce serum levels of LPS and inflammatory cytokines in NASH mice.

Currently, fewer studies have focused on the dynamic changes of the gut microbiota in the progression of NAFLD. In this study, mice were fed with the HFHC diet for 12, 18, or 24 weeks, respectively, to simulate the different stages of NAFLD, and the results showed that mice that were fed a HFHC diet for 12 weeks developed pronounced hepatic steatosis but lack of inflammation. When fed a HFHC diet for 18 weeks, hepatic lobular inflammation and ballooning degeneration, which are histopathological features of NASH, could be observed, whereas 24 weeks of HFHC diet feeding led to mild-to-moderate liver fibrosis. A recent study conducted by Boland et al. (Boland et al., 2019) showed that ob/ob mice had mild fibrosis after feeding the HFHC diet for 9 weeks, but a longer HFHC feeding period was needed in wild-type mice, which was consistent with our results. Results of bacterial 16S rRNA gene sequencing demonstrated the gut bacterial community structure was significantly drifted and evolution in HFHC-fed mice with the prolonging of the feeding period. Especially, the relative abundance of norank_f_Oscillospiraceae, Anaerostipes, and others increased or decreased in a stepwise pattern as NAFLD progressed, suggesting that these gut bacteria played a key role in the progression of NAFLD.

However, current studies about the altered gut bacteria in the development of NAFLD remain controversial. A study conducted by Shen et al. (Shen et al., 2017), including 25 NAFLD patients and 22 healthy subjects, showed that the relative abundance of Proteobacteria and Fusobacteria phyla was increased, while that of Prevotella was decreased in NAFLD patients compared to the healthy subjects. Loomba et al. enrolled 86 biopsy-proven NAFLD patients and compared the gut microbiome compositions between patients with mild-to-moderate and advanced liver fibrosis, and the results showed the abundances of Eubacterium rectale and Bacteroides vulgatus were increased, whereas the abundance of B. vulgatus and Escherichia coli were decreased in the mild-to-moderate liver fibrosis (Loomba et al., 2017). The discrepancy of the results might indicate that the diverse gut microbiota might play different roles at the different stages of NAFLD. Gut microbiota is susceptible to alterations of geographic location, environmental factors, dietary habits, age, etc., which might partially explain the discrepancy.

In this study, we also evaluated the effects of different doses of UDCA treatment on NASH and found that UDCA treatment could dose-dependently reduce serum levels of ALT and AST, which was consistent with a previous study (Buko et al., 2011). Histopathologically, the NAS scores in the HFHC-UDCA (120 mg/kg) group were significantly decreased after 4 weeks of UDCA treatment compared to the HFHC-control group. As NAS is the sum of scores for hepatic steatosis, hepatocellular ballooning, and lobular inflammation, the scores for steatosis and inflammation (hepatocellular ballooning and lobular inflammation) were compared separately among groups. Intriguingly, despite a liver weight loss, the score for hepatic steatosis was not significantly decreased in the UDCA treatment group compared to the HFHC-control group. A study conducted by Mueller et al. (Mueller et al., 2015) showed that UDCA induced the accumulation of neutral lipids in the liver *via* antagonizing farnesoid X receptor. Another RCT evaluated the efficacy of high-dose UDCA therapy for NASH and reported that UDCA only significantly improved lobular inflammation, but not steatosis and fibrosis (Leuschner et al., 2010). While several studies showed that UDCA treatment decreased hepatic triglyceride content and reversed histological steatosis (Quintero et al., 2014; Fujita et al., 2017), the different doses of UDCA across studies might partly explain the discrepancy. More importantly, in our study, the suppressed FXR signal pathway including Shp, Fxr, Klb, and Hnf4α induced by the HFHC diet was not reversed after UDCA treatment as demonstrated by PCR results. The agonistic activity on FXR was beyond the pharmacological role of UDCA (Liu et al., 2014), while obeticholic acid acting as a FXR agonist could significantly improve hepatic steatosis and increase insulin sensitivity in NASH patients (Neuschwander-Tetri et al., 2015).

Another interesting finding of the present study was that UDCA could partially restore the dysbiosis of the gut microbiota induced by the HFHC diet and repair gut barrier integrity. Since a large dose of UDCA treatment had better efficiency as demonstrated above, 16S rRNA gene sequencing on the mice gut microbiota among ND-control, HFHC-control, and HFHD-UDCA (120 mg/kg) groups was performed to analyze the gut microbial community structure, and PCoA and NMDS analyses suggested three separated clusters, indicating gut microbiota composition among the groups was different from each other. At the phylum level, Firmicutes and Bacteroidetes were two predominant phyla in our study. The HFHD diet led to an increased abundance of Firmicutes and decreased abundance of Bacteroidetes, which were considered as “fat bacteria” and “lean bacteria” (Ma et al., 2020), respectively, and such dysbiosis was reversed after UDCA treatment. As body weights and liver weights of mice in the three groups did not change significantly, UDCA treatment might, at least in part, explain the improved hepatic inflammation of NASH without diet alteration. At the genus level, the relative abundances of some gut bacteria, which are generally considered as “beneficial bacteria,” such as Bifidobacterium, Lactobacillus, and Lactococcus, were significantly decreased in the HFHC-control group, compared to the ND-control group, but did not reverse after UDCA treatment. However, the relative abundance of several intestinal bacteria that are generally considered beneficial, such as *Faecalibaculum* genus, increased in the HFHC-control group, compared to the ND-control group, and decreased after UDCA treatment. The putative cause might be that specific gut bacterial taxa play a different role in the pathogenesis of diseases. Consistent with previous studies (Rau et al., 2018), the relative abundance of Alistipes decreased in the HFHC-control group but reversed after UDCA treatment. As Alistipes is an acetate producer, which is a type of short-chain fatty acids that have anti-inflammatory actions (Leung et al., 2016), it can be suggested that improvement of hepatic inflammatory after UDCA treatment in the present study might be due to the restoration of Alistipes and its products.

In our study, we measured 4 serum cytokines, including 2 inflammatory (TNF-α and IL-1β) and 2 anti-inflammatory cytokines (IL-4 and IL-10). Interestingly, not only inflammatory cytokines (TNF-α and IL-1β) but also anti-inflammatory cytokines (IL-4 and IL-10) were increased after being fed with the HFHC diet, and decreased after the UDCA treatment. We speculated the increased levels of anti-inflammatory cytokines might be a compensatory mechanism to counteract the inflammatory reaction induced by the HFHC diet. The UDCA treatment reduced the inflammatory reaction, and then the compensatory mechanism was also downregulated correspondingly; thus, both inflammatory and anti-inflammatory cytokines decreased after the UDCA treatment.

Gut microbiota dysbiosis induced by a high-fat diet is often associated with intestinal barrier dysfunction; thus, gut-derived harmful substances including LPS, endotoxins, as well as inflammatory factors would translocate into the bloodstream through intestinal endothelial cells. In this study, immunohistochemistry staining detected significantly decreased expressions of tight junction proteins such as ZO-1 and Claudin-1, suggesting an impairment of the intestinal barrier. Moreover, increased serum levels of LPS and inflammatory factors were also observed in the mice of the HFHC-control group. LPS could activate Toll-like receptor 4 on stellate cells and Kupffer cells in the liver to stimulate the pro-inflammatory signal pathways, contributing to the progression of NAFLD. However, UDCA treatment reduced the serum levels of LPS and inflammatory factors, and increased the expressions of ZO-1 and Claudin-1 accompanied by the partial restoration of the gut microbiota. The improvement of hepatic inflammation in NASH after UDCA treatment, although could not be directly explained by, at least was associated with the restoration of the gut microbiota and intestinal barrier dysfunction. A study performed by Kim et al. showed that UDCA improved liver function in obese patients with liver dysfunction *via* microbiome remodeling (Kim et al., 2018). In another study evaluating the effect of *P. distasonis* on obesity and metabolic dysfunctions, oral administration of UDCA/LCA mixture significantly decreased hyperlipidemia and hepatic steatosis *via* repair of gut barrier integrity and activation of the FXR signal pathway (Wang et al., 2019). As UDCA is a weak ligand for FXR (Parks et al., 1999), it makes few contributions to the activation of the FXR signal pathway, but repair of gut barrier integrity.

In summary, our study provided evidence that a larger dose of UDCA is effective for treating NASH, and the proposed mechanism might be that it could partially restore gut microbiota dysbiosis and increase the expression of Claudin-1 and ZO-1 in the intestine. However, there were still some limitations in our study. First, when the dynamic change of the gut microbiota was compared among the different stages of NAFLD, we only examined the gut microbiota in the ND_12w group in the control group. It would be better that the gut microbiota in the ND_18w and ND_24w group should also be examined to exclude the effects of time changes on it. Second, although we found that UDCA could alleviate hepatic inflammation and restore gut microbiota, whether the restoration of gut microbiota is the cause of improvement of hepatic inflammation after UDCA treatment is still unknown. In our further study, we would utilize fecal microbiota transplantation to verify the causal relationship. Third, due to the limit of a research fund, only four to five mice per group were fed. Nevertheless, liver histopathology showed that all mice fed with the HFHC diet developed typical features of NASH; therefore, we believed that the results drawn from our study were convincing.

In conclusion, the present study demonstrated dynamic changes of the gut microbiota in the progression of NAFLD, which were significantly correlated with the severity of NAFLD revealed by H&E and Masson’s trichrome staining. In addition, a large dose of UDCA reduces serum levels of LPS and inflammatory cytokines through partial restoration of the gut microbiota and repair of intestinal epithelial barrier and has a beneficial effect on hepatic inflammatory in treating NASH (Figure 8B). Further studies are needed to explore the role of specific gut microbes in the pathogenesis of NAFLD.

## Data Availability

The data presented in the study are deposited in the NCBI BioProject repository, accession number PRJNA777806 and PRJNA 777878.
