# Supplementary material for: Ursodeoxycholic Acid Treatment Restores Gut Microbiota and Alleviates Liver Inflammation in Non-Alcoholic Steatohepatitic Mouse Model
Source: Front Pharmacol. 2021 Dec 6;12:788558. doi: 10.3389/fphar.2021.788558 (PMC8685972; doi:10.3389/fphar.2021.788558)
Supplement: Supplementary file 1 [file Presentation1.pdf]

Supplementary File S1: List of primers used for real-time PCR in the study.

**Genes**	**primer**	**sequence**
Cyp7α1	Forward	AGCAACTAAACAACCTGCCAGTACTA
	Reverse	GTCCGGATATTCAAGGATGCA
Cyp27α1	Forward	CCAGGCACAGGAGAGTACG
	Reverse	GGGCAAGTGCAGCACATAG
Cyp8β1	Forward	TAGCCCTCTTTCCTCCACTCAT
	Reverse	GAACCGATCGAACCTAAATTC
Ntcp	Forward	CAAACCTCAGAAGGACCAAACA
	Reverse	GTAGGAGGATTATTCCCGTTGTG
Bsep	Forward	AAGCTACATCTGCCTTAGACAC
	Reverse	CAATACAGGTCCGACCCTCTCT
Shp	Forward	TGTACACTACTAACTGTCCAGCA
	Reverse	GAGGGATTCTGGCTTGAAAGTAC
Fxr	Forward	GCTGATGTCTTGGAGAGTGAATG
	Reverse	TGAAACCAGGTACAAACGAAACA
Klb	Forward	TGTTCTGCTGCGAGCTGTTAC
	Reverse	CCGGACTCACGTACTGTTTTT
Hnf4α	Forward	CACGCGGAGGTCAAGCTAC
	Reverse	CCCAGAGATGGGAGAGGTGAT
