# Supplementary material for: Ursodeoxycholic Acid Treatment Restores Gut Microbiota and Alleviates Liver Inflammation in Non-Alcoholic Steatohepatitic Mouse Model
Source: Front Pharmacol. 2021 Dec 6;12:788558. doi: 10.3389/fphar.2021.788558 (PMC8685972; doi:10.3389/fphar.2021.788558)

Supplementary File S4: UDCA treatment does not improve the levels of serum triglycerides (A), total cholesterol (B) and blood glucose(C)

(A)

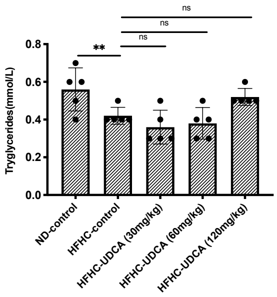

(B)

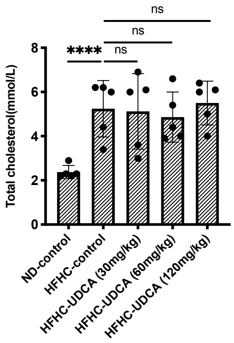

(C)

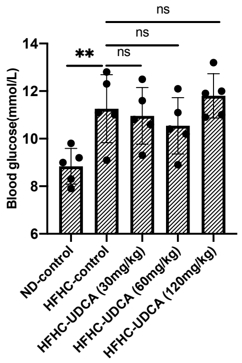

Supplement: Supplementary file 2 [file Presentation4.pdf]
