# Supplementary material for: Ursodeoxycholic Acid Treatment Restores Gut Microbiota and Alleviates Liver Inflammation in Non-Alcoholic Steatohepatitic Mouse Model
Source: Front Pharmacol. 2021 Dec 6;12:788558. doi: 10.3389/fphar.2021.788558 (PMC8685972; doi:10.3389/fphar.2021.788558)

Supplementary File S5: Rarefaction curves (A) and Shannon curves (B) of 16S rRNA sequencing of fecal microbiota from 15 mice on OUT level

(A)

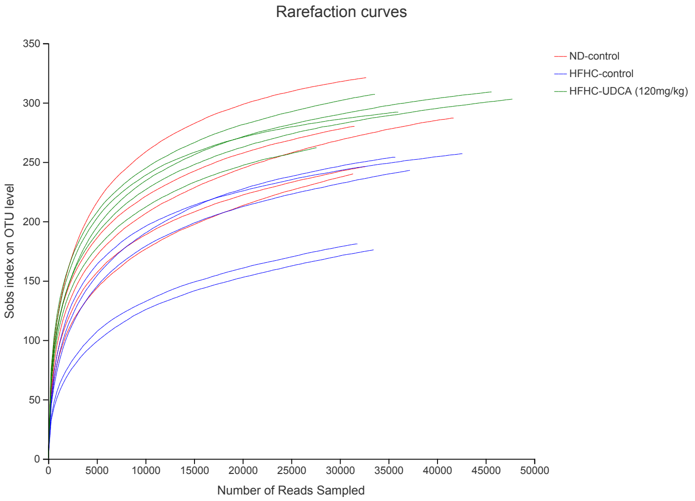

(B)

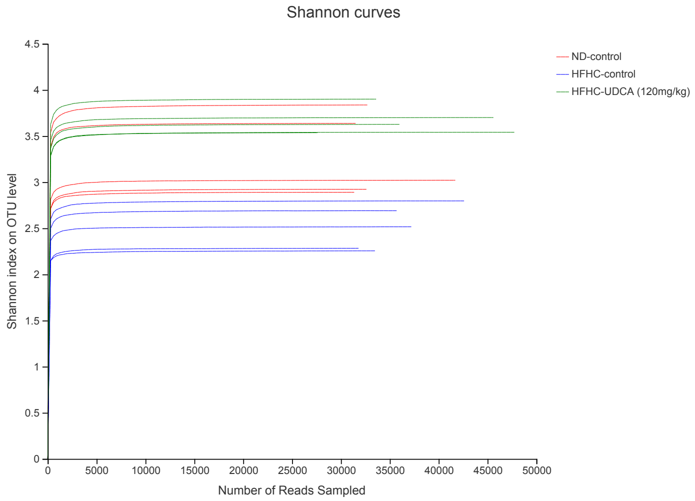

Supplement: Supplementary file 3 [file Presentation5.pdf]
