# Supplementary material for: Ursodeoxycholic Acid Treatment Restores Gut Microbiota and Alleviates Liver Inflammation in Non-Alcoholic Steatohepatitic Mouse Model
Source: Front Pharmacol. 2021 Dec 6;12:788558. doi: 10.3389/fphar.2021.788558 (PMC8685972; doi:10.3389/fphar.2021.788558)

Supplementary File S6: Representative differential pathways (A) and heatmap of all differential pathways (B) predicted by PICURSt2

(A)

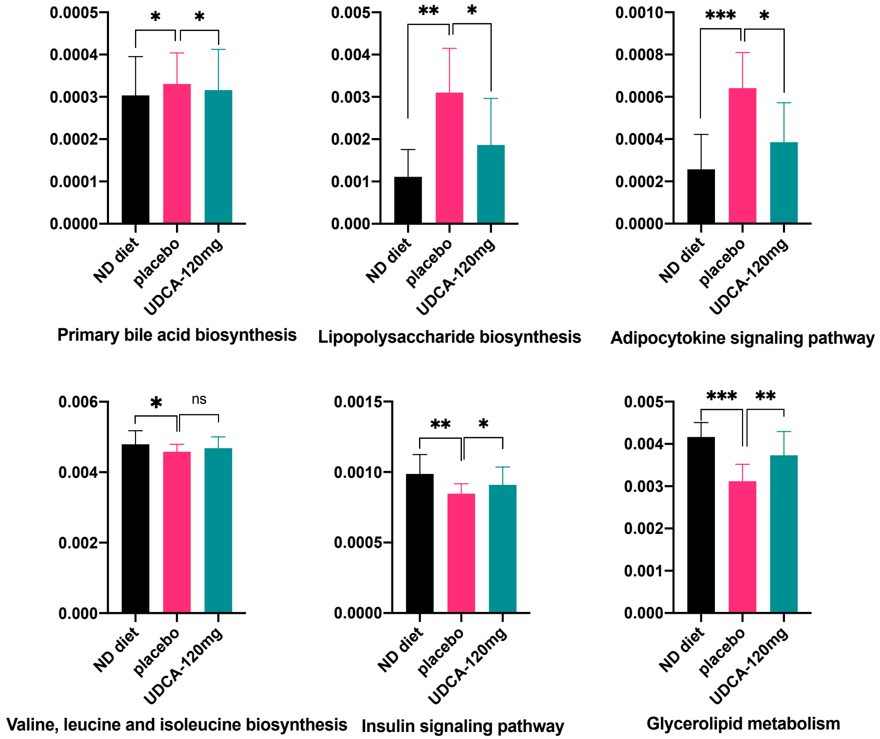

(B)

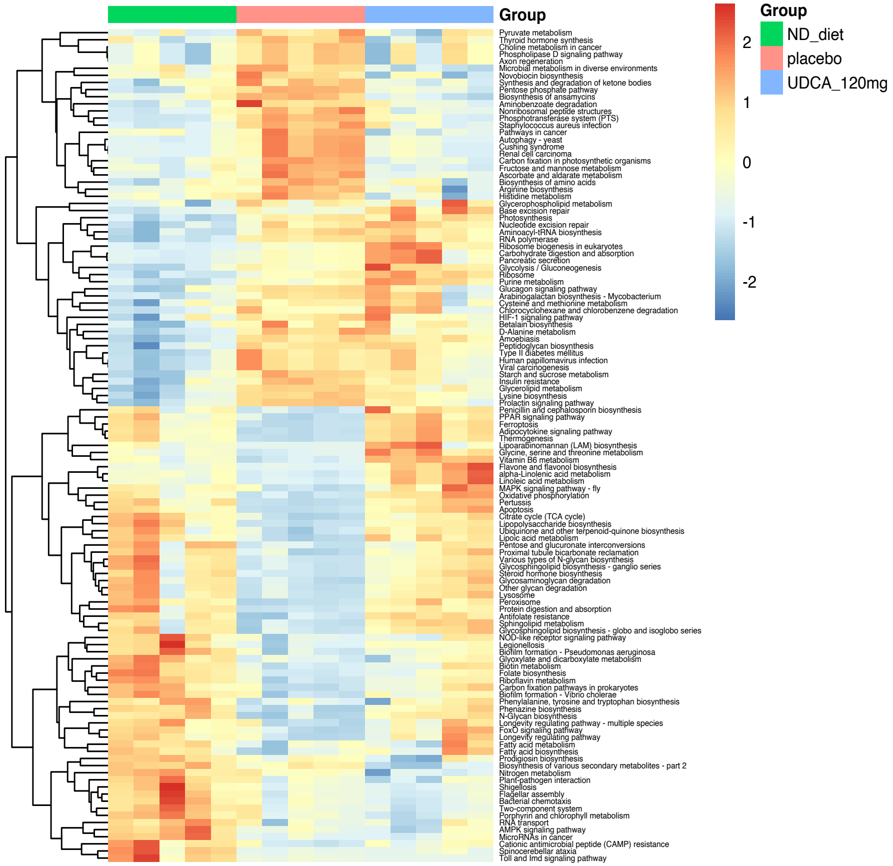

Supplement: Supplementary file 4 [file Presentation6.pdf]
