# Supplementary figures and images for: Ursodeoxycholic Acid Treatment Restores Gut Microbiota and Alleviates Liver Inflammation in Non-Alcoholic Steatohepatitic Mouse Model
Source: Front Pharmacol. 2021 Dec 6;12:788558. doi: 10.3389/fphar.2021.788558 (PMC8685972; doi:10.3389/fphar.2021.788558)

**Supplementary File S2: PCR results of extracted DNA from fecal pellets of 16 mice (A) and 15 mice(B)**

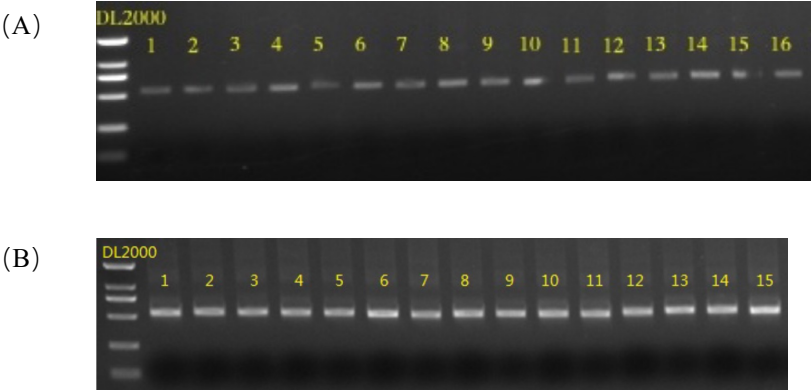

Supplement: Supplementary file 5 [file Presentation2.pdf]
